# Programmed cell death in *Helicobacter pylori* infection and related gastric cancer

**DOI:** 10.3389/fcimb.2024.1416819

**Published:** 2024-07-31

**Authors:** Yukun Lin, Kunjing Liu, Fang Lu, Changming Zhai, Fafeng Cheng

**Affiliations:** ^1^ School of Traditional Chinese Medicine, Beijing University of Chinese Medicine, Beijing, China; ^2^ School of Life Sciences, Beijing University of Chinese Medicine, Beijing, China; ^3^ Department of Rheumatism, Beijing University of Chinese Medicine Third Affiliated Hospital, Beijing, China

**Keywords:** *Helicobacter pylori*, programmed cell death, gastric cancer, infection, therapy

## Abstract

Programmed cell death (PCD) plays a crucial role in maintaining the normal structure and function of the digestive tract in the body. Infection with *Helicobacter pylori* (*H. pylori*) is an important factor leading to gastric damage, promoting the Correa cascade and accelerating the transition from gastritis to gastric cancer. Recent research has shown that several PCD signaling pathways are abnormally activated during *H. pylori* infection, and the dysfunction of PCD is thought to contribute to the development of gastric cancer and interfere with treatment. With the deepening of studies on *H. pylori* infection in terms of PCD, exploring the interaction mechanisms between *H. pylori* and the body in different PCD pathways may become an important research direction for the future treatment of *H. pylori* infection and *H. pylori*-related gastric cancer. In addition, biologically active compounds that can inhibit or induce PCD may serve as key elements for the treatment of this disease. In this review, we briefly describe the process of PCD, discuss the interaction between different PCD signaling pathways and the mechanisms of *H. pylori* infection or *H. pylori*-related gastric cancer, and summarize the active molecules that may play a therapeutic role in each PCD pathway during this process, with the expectation of providing a more comprehensive understanding of the role of PCD in *H. pylori* infection.

## Introduction

Programmed cell death is an active, inherent phenomenon. It supports normal physiological activities during development and homeostasis after birth by eliminating damaged, infected or superannuated cells. In 1972, Kerr et al. made a groundbreaking discovery by describing a phenomenon known as apoptosis (Kerr et al., 1972). This phenomenon involves a programmed condensation of the nucleus and cytoplasm, leading to fragmentation of the cell into a series of membrane-bound, structurally well-preserved fragments. Subsequently, these fragments are either shed from the epithelial-lined surfaces or taken up by other cells and rapidly degraded by lysosomal enzymes from macrophages. Subsequently, different forms of PCD were gradually discovered (**Table 1**). Today, programmed cell death includes genetically programmed suicide mechanisms, such as apoptosis, necroptosis and pyroptosis (Newton et al., 2024). It can also result from highly conserved processes to degrade macromolecular structures and even entire organelles, as in autophagy (Cadwell, 2016), or from dysregulated metabolism, as observed in ferroptosis (Tang et al., 2021).

**Table 1:** Programmed cell death pathways with their morphological and biochemical features.

Programmed cell death	Morphological features and key pathway components	References
Apoptosis	Nuclear and cytoplasmic condensation (pyknosis), formation of extracellular vesicles (apoptotic bodies) and phagocytosis by surrounding cells. Pro-apoptotic proteins of the BCL-2 family, MOMP, caspase activation (e.g. Apaf-1 in intrinsic apoptosis or FADD in extrinsic apoptosis), cleavage of caspase substrates and ROS production.	(Kerr et al., 1972) (Li et al., 1997) (Stennicke et al., 1998)
Necroptosis	Cell swelling, rupture of the plasma membrane, swelling of the cytoplasmic organelles and release of intracellular contents. Inhibition of caspase-8, activation of RIPK1 and RIPK3, formation of the necrosome complex, phosphorylation of MLKL, inflammatory response and release of DAMPs (also called PAMPs in pathogenically infected cells).	(Degterev et al., 2005) (Li and Beg, 2000) (Holler et al., 2000)
Pyroptosis	Cell swelling or lack of cell swelling, rupture of the plasma membrane and release of intracellular contents (inflammation-related). Activation of the inflammasome, cleavage of the GSDMD protein, release of pro-inflammatory cytokines (e.g. IL-18).	(Cookson and Brennan, 2001) (Moujalled et al., 2021) (Galluzzi et al., 2018)
Autophagy	Vacuolization of the cytoplasm, formation of autophagosomes, without chromatin condensation. ATG family proteins and the conversion of LC3-I to LC3-II (e.g. ATG7 and ATG3 are involved in the phosphorylation and lipidation of LC3 and promote the conversion).	(Mizushima and Komatsu, 2011) (Bergmann, 2007)
Ferroptosis	Mitochondria that appear smaller than normal, with increased membrane density. Excessive accumulation of iron, peroxidation of membrane lipids and glutathione depletion.	(Dixon et al., 2012) (Stockwell, 2022)

B-cell lymphoma gene 2 (BCL-2), Mitochondrial outer membrane permeabilization (MOMP), Apoptotic peptidase activating factor 1 (Apaf-1), FAS-associated death domain (FADD), Reactive oxygen species (ROS), Receptor-interacting serine/threonine-protein kinase 1 (RIPK1) activation, Mixed lineage kinase domain-like protein (MLKL), Damage-associated molecular patterns (DAMPs), Gasdermin D (GSDMD), Interleukin-18 (IL-18), Autophagy-related protein (ATG), Microtubule-associated protein 1A/1B-light chain 3 (LC3).

Although the PCD plays a crucial role in maintaining homeostasis in our body and keeping it in a stable state, its normal processes can be disrupted by the invasion of pathogens, especially *H. pylori* infection. This disruption can unbalance our internal environment and possibly even trigger gastric cancer (Meng et al., 2015). In the early stage of infection, *H. pylori* can induce the expression of programmed death ligand 1 (PD-L1) on the epithelial cells of the stomach, for example via the Shh signaling pathway (Holokai et al., 2019). PD-L1, which interacts with programmed death 1 (PD1) on the surface of cytotoxic T lymphocytes (CTLs), can inhibit CTLs to induce PCD (Sun et al., 2007). In this way, cells infected with *H. pylori* can be protected from the immune response. In addition, *H. pylori* infection causes visible damage to both the superficial epithelial cells and the cells that form pits and glands in the stomach, caused directly by the bacteria or by PCD-mediated mechanisms (Genta, 1997). Once the destroyed glands can no longer regenerate, fibroblasts and extracellular matrix fill the space they previously occupied in the lamina propria. This leads to an irreversible loss of functional structure known as atrophy, a condition thought to be a precursor to gastric cancer (Correa, 1995). In addition, *H. pylori* infection triggers several intracellular signaling pathways, such as mitogen-activated protein kinase (MAPK), nuclear factor kappa B (NF-κB) and phosphatidylinositol 3-kinase (PI3K). These signaling pathways in turn have a direct and indirect effect on the proliferation, differentiation and programmed cell death of the gastric epithelium and ultimately lead to the conversion of epithelial cells into oncogenic units (Kato et al., 2008; Yousefi et al., 2019).

Overall, it is clear that PCD plays an important role in *H. pylori* infection. However, the relationship between PCD and *H. pylori* infection is complicated and not simply linear. During the infection process, *H. pylori* can both promote and inhibit PCD. For example, *H. pylori* infection can trigger both ROS formation and DNA fragmentation, leading to the activation of caspase-3 and caspase-8. This suggests that oxidative stress can be exerted on gastric epithelial cells during *H. pylori* infection, leading to apoptosis (Ding et al., 2007). However, other studies have shown that the gamma-glutamyl transpeptidase (GGT) of *H. pylori* can inhibit apoptosis and induce proliferation of gastric epithelial cells through the induction of cyclooxygenase-2, epidermal growth factor-related peptides and interleukin-8 (Ricci et al., 2014).

In this review, we provide a brief overview of the different pathways of PCD, including apoptosis, necroptosis, pyroptosis, autophagy and ferroptosis. We discuss their known and proposed roles in *H. pylori* infection to elucidate the intricate relationships. In addition, we describe proposed therapeutic strategies for the treatment of *H. pylori* infection that target key regulators of various PCD signaling pathways.

## Programmed cell death in *H. pylori* infection and related gastric cancer

### Apoptosis, common in *H. pylori* infection

The term “apoptosis” comes from the Greek and means “falling away”, which reflects the characteristic property of apoptotic cells to disintegrate and fragment in a controlled manner. At the molecular level, apoptosis is triggered by either intrinsic or extrinsic signaling pathways, both of which converge in a common execution phase (Green, 2000). Intrinsic apoptosis is triggered by intracellular signals such as DNA damage, oxidative stress or growth factor deficiency and leads to the activation of pro-apoptotic proteins of the BCL-2 family, including BAX and BAK (Puthalakath and Strasser, 2002). These proteins promote permeabilization of the outer mitochondrial membrane, leading to the release of cytochrome c into the cytoplasm (Jost and Vucic, 2020). Cytochrome c then activates the caspase cascades, which ultimately leads to cell death. Extrinsic apoptosis, on the other hand, is triggered by extracellular signals, e.g. by the binding of death ligands to death receptors on the cell surface, such as tumor necrosis factor receptor 1 (TNFR1) or FAS. This interaction recruits and activates caspase-8, which can directly cleave and activate downstream effector caspases or trigger the mitochondrial signaling pathway through BID cleavage (Czabotar et al., 2014).

**Figure 1:**
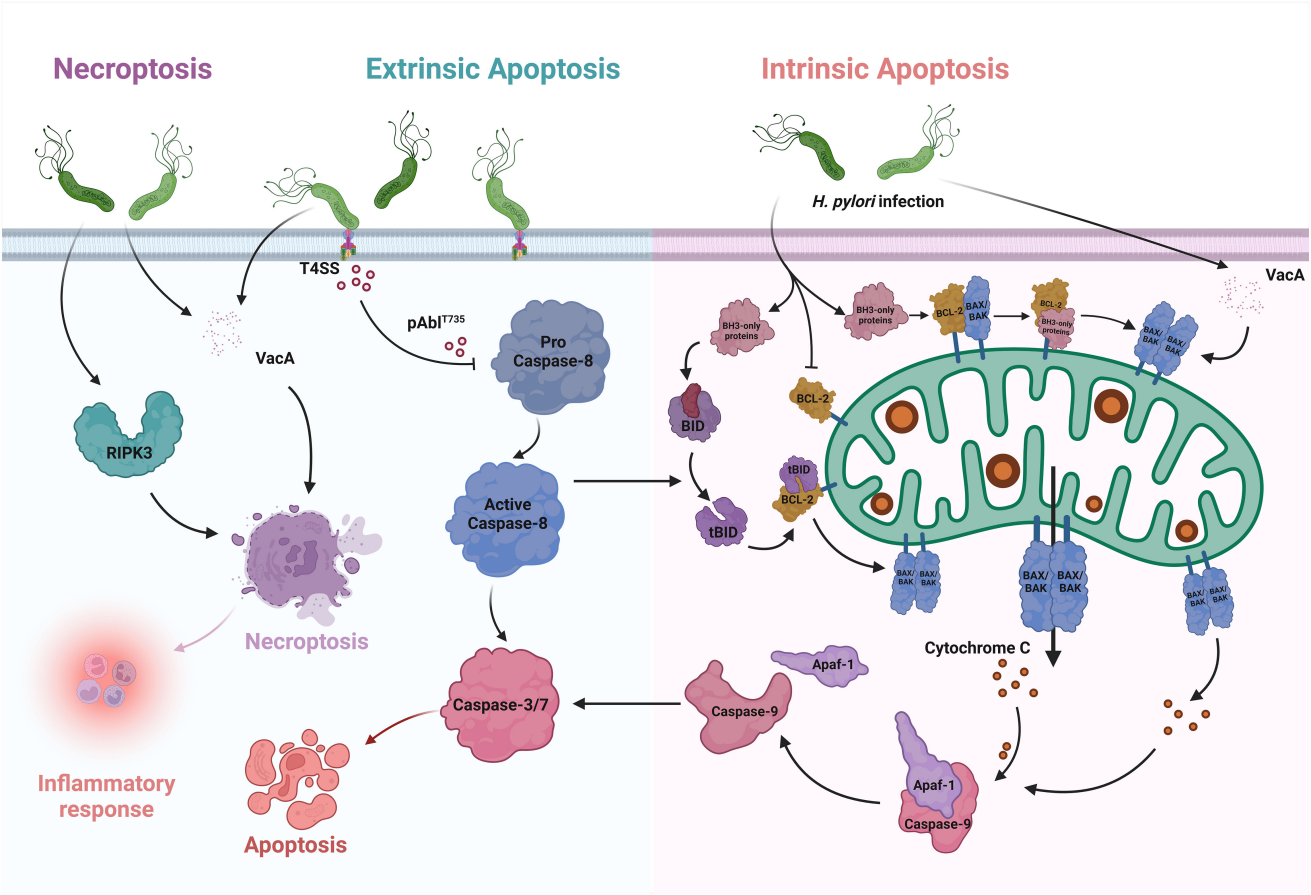
Apoptosis and Necroptosis in *H. pylori* infection. In the intrinsic pathway of apoptosis, only BH3 proteins are upregulated after *H. pylori* infection. They interact with the BCL-2 proteins, which are essential for survival, and thus override the inhibition of BAX and BAK. As a result, BAX and BAK oligomerize, leading to MOMP. After its release into the cytoplasm, cytochrome c binds to Apaf-1, which activates the initiator caspases and induces apoptosis. VacA, which causes the translocation of BAX and increases cytochrome c, can also enhance apoptosis. BID is one of the pure BH3 proteins. It can be cleaved by activated caspase-8 and releases BAX and BAK to activate intrinsic apoptosis via the MOMP pathway. In the extrinsic pathway, *H. pylori* promotes the formation of pAbIT735 via the type IV secretion system (T4SS), thereby attenuating caspase-8-dependent cell apoptosis. In necroptosis, infection with *H. pylori* leads to an increase in the key factor RIPK3, which promotes the occurrence of inflammatory reactions. VacA also triggers necroptosis and releases considerable amounts of inflammatory mediators.

With the exponential increase in the number of publications on *H. pylori* as well as those written on apoptosis, it is clear that apoptosis is closely intertwined with *H. pylori* infection (Shirin and Moss, 1998). In the context of infection, there are changes in the extent of cell apoptosis, accompanied by changes in cell proliferation, migration and cytokine production (Alzahrani et al., 2014). In addition to directly triggering apoptosis, *H. pylori* induces sensitivity to various epithelial signaling pathways and events in the host. Certain mechanisms are triggered by *H. pylori* binding to cell surface receptors or by soluble virulence factors entering the epithelium, leading to the initiation of gastric pathologies, including inflammation, mucosal damage, and even the development of gastric cancer (**Figure 1**) (Tsai and Hsu, 2017).

### Different apoptotic effects in *H. pylori* infection

Infection with *H. pylori* has different apoptotic effects on different types of host cells. In most cells, *H. pylori* infection increases apoptosis. In gastric epithelial cells, acute *H. pylori* infection significantly accelerated GES-1 apoptosis by increasing the expression of BAX and cleaved caspase-3, while decreasing the expression of BCL-2 (Liu et al., 2021). In lymphocytes, the researchers observed the transfer of apoptosis-inducing factor (AIF) from the mitochondria to the nucleus. In addition, apoptosis of both T and B cells was significantly increased in *H. pylori* infected cells compared to uninfected controls (Singh et al., 2006). *H. pylori* induces apoptosis in T and B cell lines and translocates AIF. The destruction of T and B cells by apoptosis could explain the persistence of *H. pylori* infection. In the mononuclear phagocyte system, macrophages under infection have also shown the ability to induce apoptosis by the protein HP1286 secreted by *H. pylori* (Tavares and Pathak, 2017). Co-culturing *H. pylori* with peripheral blood monocytes, THP-1 or U937 cells leads to increased apoptosis (Zhang et al., 2017a). However, the researchers found that suppressing the expression of leukocyte-associated immunoglobulin-like receptor-1 (LAIR-1) on THP-1 cells can reduce this process (Zhang et al., 2017b). The results suggest that LAIR-1 modulates cell apoptosis and secretion of inflammatory cytokines in THP-1 cells, which may help to maintain inflammation and prevent the clearance of bacteria by the immune response. In gastric cancer cells, infection with *H. pylori* resulted in cell cycle arrest, decreased proliferation and increased apoptosis (Ding et al., 2008). *H. pylori* increased apoptosis in AGS cells with chromatin condensation and decreased BCL-2 levels, which was associated with NF-κB activation (Chu et al., 2003). In addition, *H. pylori* induced cullin 1-RING ubiquitin ligase (CRL1) and 26S proteasome-dependent degradation of STAMBPL1 in AGS (Chaithongyot and Naumann, 2022). STAMBPL1, which functions as a deubiquitinase of the anti-apoptotic protein survivin, balances the extent of survivin degradation with the E3 ligase CRL1 and thus regulates apoptotic cell death. Thus, the degradation of STAMBPL1 promotes apoptotic cell death.

However, there are always exceptions. Yang et al. found that *H. pylori* infection promotes apoptosis of MGC-803 cells, but high expression of FRA-1 induced by *H. pylori* suppresses this cell death (Yang et al., 2021). Liu et al. showed that acute infection with *H. pylori* significantly accelerated the apoptosis of GES-1, but the apoptosis rate of GES-1 cells with chronic *H. pylori* infection decreased significantly (Liu et al., 2021). Zhang et al. also demonstrated a novel mechanism by which *H. pylori* escapes from monocytes by upregulating early apoptosis and inhibiting late apoptosis (Zhang et al., 2017a). These studies have shown that inhibition of apoptosis of gastric epithelial cells due to chronic *H. pylori* infection is a contributing factor to severe gastric diseases, leading us to realize that the influence of *H. pylori* on cell apoptosis may not be unidirectional. An imbalance between apoptosis and proliferation may contribute to *H. pylori*-associated gastric carcinogenesis. Clinical research has also confirmed that dysregulation of apoptosis control in gastric intestinal metaplasia could further exacerbate gastric carcinogenesis, while elimination of *H. pylori* could potentially delay this process (Leung et al., 2001). It is worth noting that cell escape from apoptosis is an important link in the process of carcinogenesis.

### Key virulence factors in apoptosis

*H. pylori* affects apoptosis through several virulence factors (Gonciarz et al., 2019). Among the numerous factors, the importance of Vacuolating cytotoxin A (VacA) and Cytotoxin-associated gene A (CagA) is obvious. Clinical studies have shown that gastric mucosal proliferation significantly correlates with the severity of acute gastritis in individuals infected with CagA+ VacA s1a strains of *H. pylori*. However, this increased proliferation was not accompanied by a parallel increase in apoptosis (Peek et al., 1997). The increased cell proliferation in the absence of a corresponding increase in apoptosis may explain the increased risk of gastric carcinoma associated with infection by CagA+ VacA s1a strains of *H. pylori*. VacA is observed in almost all clinical strains of *H. pylori*. While only certain strains produce the toxic and pathogenic VacA, this variant can induce vacuolization and apoptosis of cells (Ansari and Yamaoka, 2020). VacA can affect immune cells such as T cells and dendritic cells, leading to increased cell apoptosis (Winter et al., 2014; Kim et al., 2015). In addition, VacA can induce apoptosis in gastric cancer cells such as AGS and AZ-521 via low-density lipoprotein receptor-related protein-1 (LRP1) (Yahiro et al., 2012). But in some situations, the effect of VacA on cell apoptosis is also dual. In the response to *H. pylori* infection, infiltration of numerous inflammatory cells such as eosinophils occurs. VacA caused the translocation of cytoplasmic BAX to mitochondria and increased cytochrome c to facilitate apoptosis, while the expression of cellular apoptosis inhibitory protein (c-IAP)-2 was upregulated in the early phase of VacA stimulation (Kim et al., 2010). Another important virulence factor of *H. pylori* is the Cag secretion system. This system translocates CagA and peptidoglycan into the host cells, leading to the activation of PI3K signaling pathways. Activation of PI3K attenuated apoptosis in response to infection and was required for cell migration induced by *H. pylori* (Nagy et al., 2009). CagA can also inhibit cell apoptosis by reducing the expression of the tumor-suppressive E3 ubiquitin ligase proteins SIVA1 and ULF in SNU1 cells, possibly promoting the development of gastric cancer (Palrasu et al., 2022).

Having discussed the main virulence factors in *H. pylori* infection, we must not overlook other factors. *H. pylori* outer inflammatory protein A (OipA) is an outer membrane protein that contributes to gastric inflammation. It can trigger toxic events and initiate the apoptotic cascade in AGS through the intrinsic pathway (Teymournejad et al., 2017). In addition, Zhao et al. have shown that OipA affects apoptosis and cell cycle of AGS cells independent of its gene copy number (Zhao et al., 2020). However, Al-Maleki et al. reported that OipA “off” and ΔOipA cause a higher level of apoptosis in AGS cells than OipA “on” strains, and deletion of OipA increased bacterial VacA production (Al-Maleki et al., 2017). Virulence factors secreted by *H. pylori*, such as lipopolysaccharide (LPS) and the antigen complex glycic acid extract (GE), are also responsible for triggering apoptosis in gastric epithelial cells (Piotrowski et al., 1997; Gonciarz et al., 2020). In addition, the *H. pylori* T4SS effector D-glycero-β-D-manno-heptose-1,7-bisphosphate (βHBP) can trigger strong c-Abl threonine 735 phosphorylation and the process attenuates extrinsic apoptosis (Posselt et al., 2019). All these reports suggest that various virulence factors produced by *H. pylori* can regulate apoptosis through multiple signaling pathways. In addition to the bacterial virulence factors, the outer membrane vesicles (OMVs) secreted by *H. pylori*, which are involved in the transport of these factors, can also influence apoptosis through their various biologically active compounds (Chmiela et al., 2018). These metabolic by-products of *H. pylori* affect the migration, proliferation and apoptosis of normal gastric cells, while they do not affect the proliferation and migration of gastric cancer cells (He et al., 2020). This creates the conditions for the transition from gastritis to gastric cancer under *H. pylori* infection.

### Dysregulation of apoptosis, from infection to gastric cancer

Apoptosis triggered by *H. pylori* may play a key role in the development of gastric cancer (Xia and Talley, 2001). Dysregulation of apoptosis within the gastric epithelium and the surrounding microenvironment is a characteristic feature of *H. pylori* infection and contributes to the development of gastric cancer (Lim et al., 2023). *H. pylori* leads to chronic inflammation because the host is unable to eradicate the infection. The chronic inflammation leads to oxidative stress originating from immune cells and within the epithelial cells of the stomach (Hardbower et al., 2013). *H. pylori* infection can synergistically interact with the tumor microenvironment (TME) and lead to DNA damage, abnormal gene expression and activation of signaling pathways. In addition, it affects the host immune system to promote tumor cell proliferation and metastasis, facilitate epithelial-mesenchymal transition (EMT), suppress apoptosis, and provide energy for tumor growth (Zhu et al., 2022). EMT is the most important biological event in epithelial cell invasion or metastasis. Yu et al. investigated that *H. pylori* CagA also triggers EMT in gastric cancer cells and promotes the mobility of gastric cancer cells by regulating PDCD4 (Yu et al., 2014).

**Figure 2:**
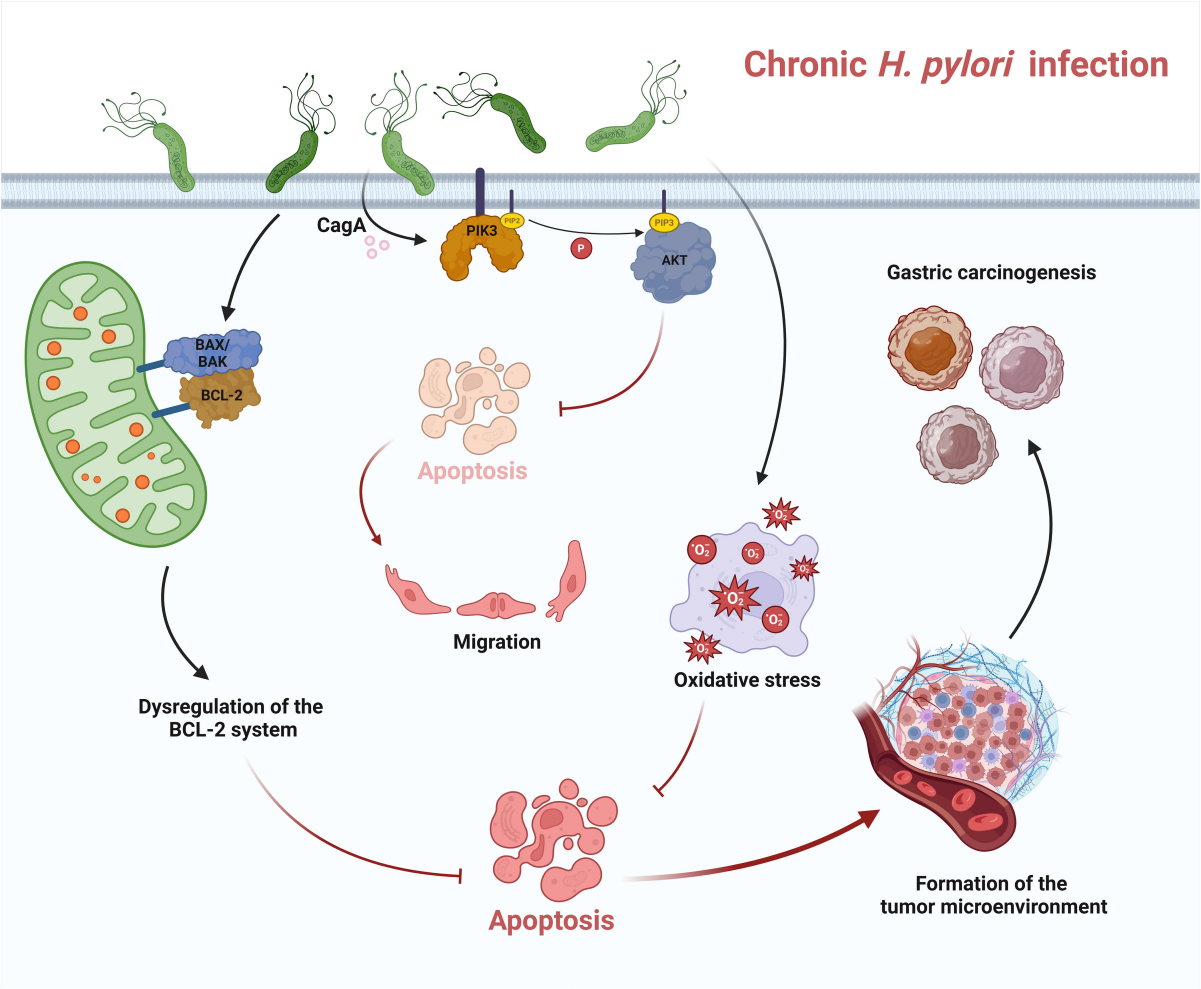
Apoptosis in chronic *H. pylori* infection. In chronic infection, *H. pylori* can trigger dysregulation of the BCL-2 system and downregulate apoptosis. *H. pylori* also leads to chronic inflammation, triggers oxidative stress and inhibits apoptosis. CagA, the virulence factor released by *H. pylori*, can regulate the activation of PI3K and AKT. AKT-dependent phosphorylation of caspase-9 attenuates apoptosis. All these dysregulations of apoptosis in chronic infection lead to the formation of a tumor microenvironment and ultimately contribute to the development of gastric cancer.

The pathways by which *H. pylori* triggers carcinogenesis and progression of gastric cancer are complex and include nitration and oxidation of DNA by mutagenic factors, epigenetic alterations by *H. pylori*, disruption of the balance between cell proliferation and apoptosis, and promotion of cancer cell invasion and metastasis by *H. pylori* (Meng et al., 2015). A clinical report showed that dysregulation of the BAX/BCL-2 system was observed in gastric cancer with a significant down-regulation of the pro-apoptotic effect. This uncontrolled cell proliferation is coupled with various proto-oncogenes that contribute to this process, resulting in inhibition of apoptosis and increased cell survival, ultimately promoting tumor growth (**Figure 2**) (Konturek et al., 2001). Rosania et al. also reported that BCL-2 gene expression decreased in preneoplastic gastric lesions such as atrophic gastritis and intestinal metaplasia. However, they found that proliferation and apoptosis did not correlate with the status of *H. pylori* infection (Rosania et al., 2017).

### Necroptosis in *H. pylori* infection

Necroptosis is a form of PCD that differs from apoptosis in both its morphological and biochemical characteristics. While apoptosis is generally considered to be a process involving controlled shrinkage and fragmentation of the cell, necroptosis is a programmed form of necrosis leading to cell swelling, rupture of the plasma membrane and release of cell contents, which in turn results in inflammation and immune activation (Degterev et al., 2005). At the molecular level, necroptosis is initiated by the activation of RIPKs, in particular RIPK1 and RIPK3 (Sun et al., 2012). During necroptosis, these kinases form a complex known as the necrosome, which phosphorylates and activates the pseudokinase MLKL. The activated MLKL oligomerizes and translocates to the plasma membrane, where it disrupts membrane integrity and leads to cell lysis (Murphy et al., 2013). Necroptosis can be triggered in response to various stimuli, including death receptor activation, toll-like receptor signaling, or cellular stress such as DNA damage or viral infection (Mocarski et al., 2011). In contrast to apoptosis, which is often inhibited by the activation of caspases, necroptosis can occur when caspase-8 is blocked, making it a potential surrogate mechanism for cell death in conditions where apoptosis is impaired (Tummers and Green, 2017).

Signs of necroptosis were found in *H. pylori* infection. Virulence factors, such as VacA, induce necroptosis in immune cells and release high levels of inflammatory mediators. In the tissues of patients with clinical *H. pylori* infection, increased expression of the necroptosis key factor RIPK3-positive cells was found in both gastritis and atrophic lesions (**Figure 1**) (Cui et al., 2022). Phenotypic analysis showed that numerous RIPK3-positive cells in the gastric glands were identified as H + K+ ATPase-positive parietal cells, while in the lamina propria they were mainly CD3-positive T lymphocytes and CD68-positive macrophages.

### Pyroptosis dysregulation in *H. pylori* infection

Pyroptosis is a form of PCD characterized by inflammatory reactions and cell lysis. It is distinct from apoptosis and necroptosis and plays a crucial role in the defense against microbial infections as well as in various inflammatory diseases (Pachathundikandi et al., 2016). Pyroptosis is triggered by the activation of specific intracellular signaling pathways that lead to the formation of large pores in the plasma membrane and subsequent cell swelling and lysis (Cookson and Brennan, 2001). At the molecular level, pyroptosis is mainly mediated by a group of proteins known as inflammasomes. These are intracellular multiprotein complexes that recognize microbial infections or cell damage (Martinon et al., 2002). In response to these stimuli, the inflammasomes assemble and activate caspase-1, also known as interleukin-1β (IL-1β)-converting enzyme, which cleaves GSDMD, a key executioner protein of pyroptosis (Shi et al., 2015). Cleaved GSDMD forms pores in the plasma membrane, leading to osmotic swelling and eventual cell lysis. The activation of inflammasomes can be triggered by various DAMPs (also called PAMPs in pathogen-infected cells) that originate from microbial infections or host cells (Rathinam et al., 2010). These include bacterial LPS, bacterial DNA and host-derived molecules such as ATP and uric acid crystals (Kayagaki et al., 2013). When these signals are sensed, oligomerization of inflammasomes occurs, particularly the NLRP3 inflammasome (Nucleotide-Binding Oligomerization Domain-like Receptor Family, Pyrin Domain-Containing 3), which recruits the adaptor protein ASC and leads to activation of caspase-1 and subsequent pyroptosis (Pachathundikandi et al., 2020). Pyroptosis is associated with the release of pro-inflammatory cytokines, including IL-1β and IL-18, which are synthesized as inactive precursors and require cleavage by activated caspase-1 for activation and secretion. The release of these cytokines amplifies the inflammatory response, recruits immune cells to the site of infection and helps eliminate pathogens (Galluzzi et al., 2018).

**Figure 3:**
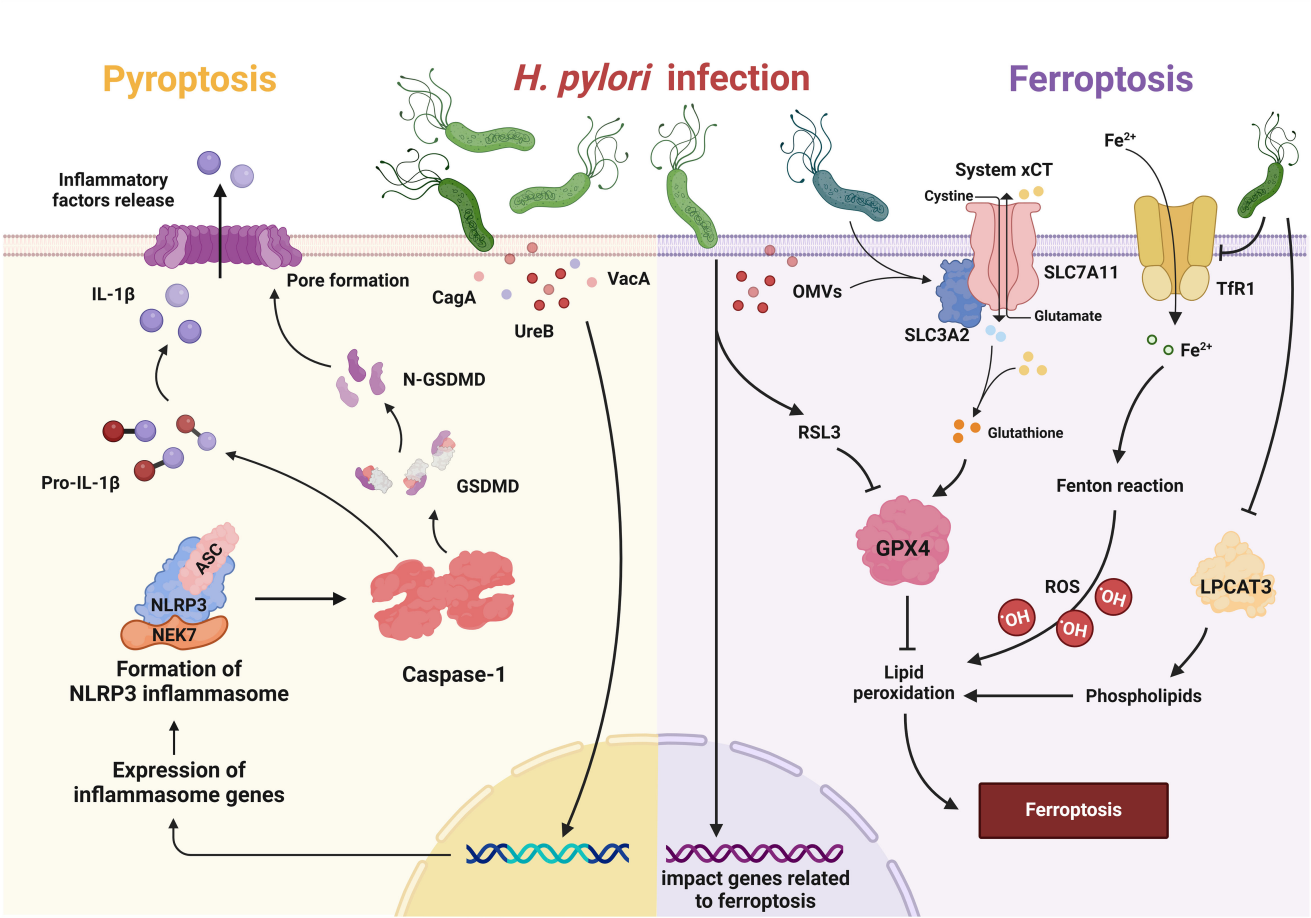
Pyroptosis and ferroptosis in *H. pylori* infection. In pyroptosis, *H. pylori* and its virulence factors (e.g. CagA, VacA, UreB) trigger the inflammatory cascade of NLRP3, which leads to the activation of caspase-1. Caspase-1 cleaves pro-IL-1β to generate its active form and also targets GSDMD, leading to recruitment of the N-terminal fragment of GSDMD to the plasma membrane. This leads to pore formation and the subsequent release of inflammatory factors. Upon ferroptosis, *H. pylori* and its OMVs upregulate Solute Carrier Family 3 Member 2 (SLC3A2) to attenuate ferroptosis, while repressing lysophosphatidylcholine acyltransferase 3 (LPCAT3) and downregulating transferrin receptor 1 (TfR1) to attenuate lipid peroxidation. *H. pylori* may also increase susceptibility to RAS-selective lethality 3 (RSL3)-induced ferroptosis by influencing associated genes.

Pyroptosis dysregulation is associated with *H. pylori* infection. *H. pylori* can regulate the key steps of pyroptosis through various virulence factors such as UreB, CagA and VacA, thereby initiating the inflammatory cascade (**Figure 3**) (Kumar and Dhiman, 2018). Although various virulence factors can trigger pyroptosis, the flagellin protein of *H. pylori*, which can trigger NLRC4 phosphorylation, does not activate the inflammasome, suggesting that NLRC4 phosphorylation is not sufficient for inflammasome activation (Matusiak et al., 2015). These results were supported by the observation that S533 is phosphorylated in the inactive NLRC4 monomer (Hu et al., 2013).

### The NLRP3 inflammasome

In *H. pylori* infection, IL-1β is highly expressed, leading to gastric acid inhibition, gastric cancer-associated gene methylation and angiogenesis. The NLRP3 inflammasome facilitates the maturation of IL-1β in various cell types such as macrophages, neutrophils and dendritic cells (Yuan et al., 2023). Another study showed that *H. pylori* infection indeed upregulates the expression of pro-IL-1β in human immune cells, but secretes only very low levels of mature IL-1β. However, administration of exogenous control activators such as nigericin or ATP to infected cells immediately triggered the formation of the NLRP3 inflammasome and the subsequent release of substantial amounts of mature IL-1β (Pachathundikandi et al., 2020). This suggests that chronic *H. pylori* infection manipulates inflammasome activation and pyroptosis to promote bacterial persistence.

Nevertheless, this deregulation of the inflammasome during *H. pylori* infection is susceptible to external stimulation by microbial, environmental, or host molecules that act as inflammasome activators, leading to the production of large amounts of mature IL-1β and signal-mediated gastric tumorigenesis in humans. Zhang et al. found that *H. pylori* infection leads to NLRP3 inflammasome activation, generation of intracellular ROS, and increased gastric cancer cell invasion and migration. In addition, ROS inhibition by N-acetyl-L-cysteine (NAC) effectively blocks NLRP3 activation and pyroptosis. Silencing of NLRP3 reduces the effects of *H. pylori* infection on gastric cancer cell migration and invasion (Zhang et al., 2022a). Taken together, this suggests that the role of the NLRP3 inflammasome in *H. pylori* infection deserves attention.

## Programmed cell death associated with autophagy

Autophagy, derived from the Greek words “auto” meaning self and “phagy” meaning to eat, is a highly conserved cellular process that is essential for maintaining cellular health and homeostasis (Ohsumi, 2014). It plays a crucial role in various physiological processes such as development, immunity and energy metabolism, and its dysregulation has been found in numerous diseases such as cancer, neurodegenerative disorders and metabolic syndromes. The process of autophagy begins with the formation of a double-membrane structure, the autophagosome. This structure engulfs cellular components to be degraded, such as damaged organelles or protein aggregates. The autophagosome then fuses with lysosomes, organelles filled with digestive enzymes, to form an autolysosome. In the autolysosome, the engulfed cargo is broken down into its individual molecules such as amino acids, fatty acids and sugars, which can then be recycled by the cell to generate energy or build new cell components (Zhang et al., 2014). There are three different types of autophagy, each of which fulfills different functions within the cell. Macroautophagy is the best-studied form of autophagy and involves the massive degradation of cytoplasmic contents. In microautophagy, cytoplasmic components are taken up directly by the lysosomes by invagination of the lysosomal membrane. Chaperone-mediated autophagy (CMA) selectively targets specific proteins for degradation by directing them to the lysosomes with the help of chaperone proteins (Yang and Klionsky, 2020). Under normal circumstances, autophagy can be regarded as a PCD process in cells or organelles (Chesnokov et al., 2024). It is a renewal process in which cellular components are recycled and redistributed to enable a dynamic balance within the cell. It is like tearing down an old wall and using the bricks to build a new house. When autophagy is disrupted, impaired cellular components can aggregate, leading to cellular senescence or autophagic cell death (Nabavi-Rad et al., 2023). Normally, autophagy turns off apoptosis, while activation of pro-apoptotic caspases can interrupt the autophagy process. However, under certain circumstances, autophagy can consume too much cytoplasmic material and promote apoptosis or necrosis, ultimately leading to autophagic cell death (Mariño et al., 2014). Unfortunately, *H. pylori* infection can promote autophagic dysregulation.

### Autophagy and *H. pylori* infection

Infection with *H. pylori* has different autophagic effects on various types of host cells. In macrophages, *H. pylori* can secrete cholesterol-α-glucosyltransferase (CGT), which inhibits the fusion of autophagosomes with lysosomes, leading to a significant increase in bacterial load within macrophages, thereby impairing the autophagic process of macrophage clearance (Lai et al., 2018). In gastric epithelial cells, *H. pylori* manipulates the NOX-ROS-Nrf2/HO-1-ROS loop to control intracellular oxidative stress and also affects ROS-mediated autophagy (Li et al., 2023a). In gastric cancer cells, autophagy is significantly altered after *H. pylori* infection and dysregulation of autophagy may be a causative factor for promoting the production of pro-inflammatory mediators in the human body (Halder et al., 2015; Sakatani et al., 2023). Initially, autophagy is a crucial pathway for controlling infection. However, prolonged exposure of the cells to the toxin VacA disrupts the induction of autophagy. This loss of autophagy leads to an accumulation of ROS, which can exacerbate inflammation and eventually lead to carcinogenesis (Raju et al., 2012). In *H. pylori* infection, the regulation of cellular autophagy can be mediated by microRNAs. Tang et al. showed that MIR30B was upregulated during *H. pylori* infection and favored bacterial replication by directly targeting ATG12 and Beclin1, important proteins involved in autophagy (Tang et al., 2012). Impairment of autophagy by MIR30B allows intracellular *H. pylori* to evade autophagic clearance and thus promotes the persistence of *H. pylori* infection. In another study, a significant correlation was found between MIR155 and immunohistochemical grade in *H. pylori*-positive patients. High expression of MIR155 could significantly reduce *H. pylori* survival by inducing autophagy (Wu et al., 2016). However, too much of a good thing is not always good. The increase of autophagy mediated by the Nrf2-HO-1 axis plays an important role in promoting *H. pylori* induced gastric carcinogenesis (Paik et al., 2019). VacA in *H. pylori* can also induce autophagy by promoting the formation of autophagosomes (Terebiznik et al., 2009). It can impair the activity of the lysosomal calcium channel MCOLN1/TRPML1, leading to the formation of enlarged, dysfunctional lysosomes and autophagosomes (Capurro et al., 2020). These autophagosomes are distinct from the induced large vacuoles and serve as an intracellular niche that allows the bacteria to evade eradication therapy. At this point, inhibition of autophagy stabilizes VacA and reduces vacuolization in the cells, limiting the damage caused by the toxin to the host cells (Raju and Jones, 2010). Thus, when autophagy is weakened, *H. pylori* can proliferate in the human body and its toxins cannot be effectively eliminated. Conversely, when autophagy is strengthened, *H. pylori* can also hide in abnormally increased autophagosomes and thus escape elimination by the host organism. It appears that *H. pylori* interferes with the normal autophagy process independently of the changes in autophagy and thus triggers negative reactions in the body. Furthermore, since there is evidence that autophagy associated with *H. pylori* depends on host cell types and bacterial strains, the ability of *H. pylori* to trigger autophagic responses should not be generalized (Deen et al., 2013).

### Key virulence factors in autophagy

**Figure 4:**
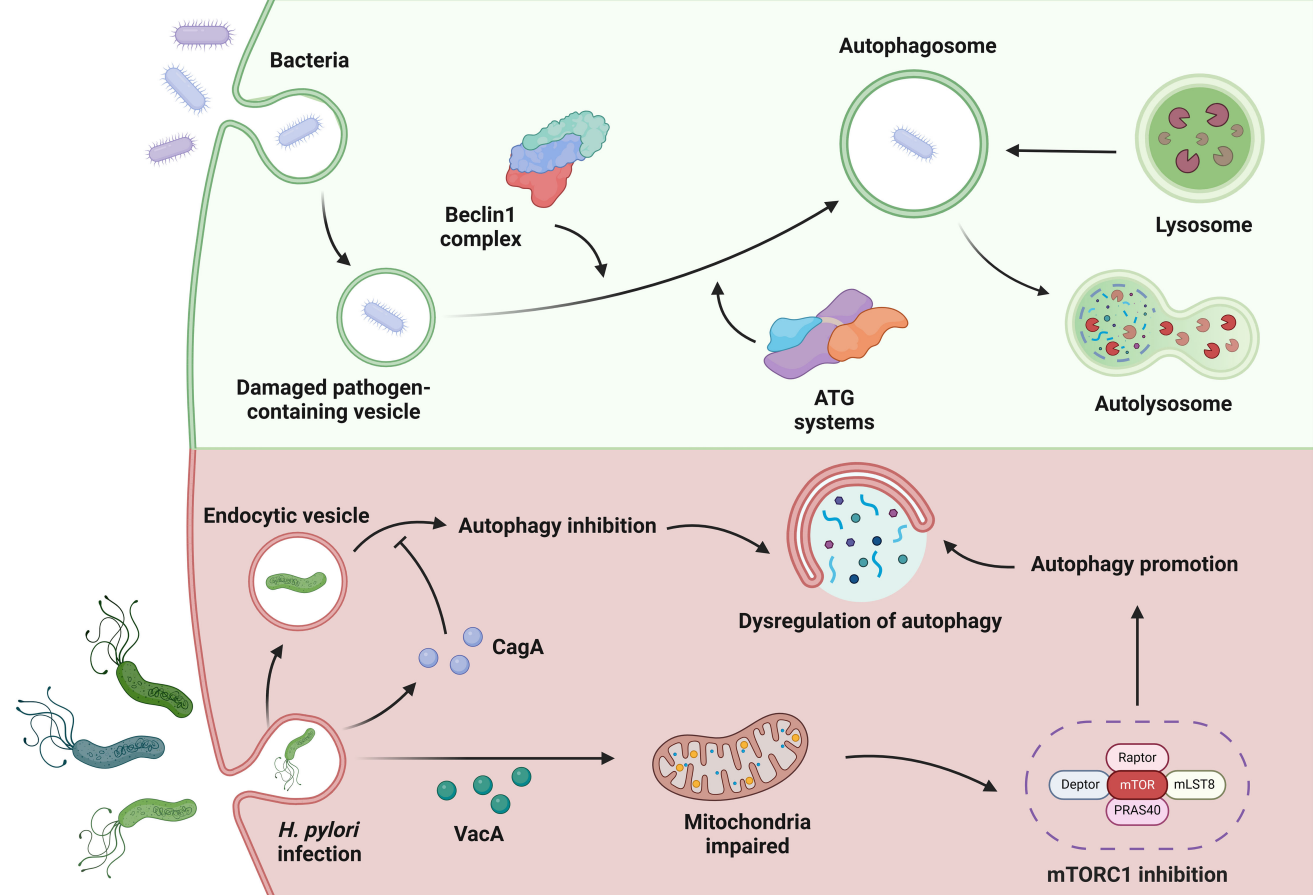
Autophagy in *H. pylori* infection. In autophagy, bacteria can be used to form damaged pathogen-containing vesicles. Under the action of the Beclin1 complex and the ATG systems, autophagosomes are formed that cooperate with the lysosomes in the cell to complete autophagy. When infected with *H. pylori*, the bacterial toxin VacA can target mitochondria to inhibit mTORC1 to promote cell autophagy. CagA, another toxin factor secreted by *H. pylori*, can evade autophagic degradation and gradually reduce cell autophagy. These complex circumstances during *H. pylori* infection lead to dysregulation of autophagy.

While the pathogenic mechanisms of *H. pylori* and its virulence factors are diverse, VacA and CagA play a crucial role in the interaction between *H. pylori* and the host autophagic machinery (**Figure 4**). VacA plays a key role in the pathogenesis of the disease by exerting pleiotropic effects on the host (Greenfield and Jones, 2013). One effect of acute VacA exposure is the induction of autophagy. However, prolonged exposure to the toxin impedes autophagy by inhibiting the maturation of autolysosomes. Kim et al. reported that the mitochondria-targeting bacterial toxin VacA inhibits a key sensor of host nutritional status, the mammalian target of rapamycin complex 1 (mTORC1), leading to a general cellular shift from biosynthetic to catabolic metabolism and further triggering the autophagic response (Kim et al., 2018). CagA can evade autophagic degradation in the host cells and thus exert its toxic effect (Tsugawa et al., 2019). Xie et al. found that autophagy initially increased and then gradually decreased during the duration of *H. pylori* infection *in vitro*, in a CagA-dependent manner. Moreover, dysregulation of autophagy promoted DNA damage in *H. pylori*-infected cells (Xie et al., 2020).

### Dysregulation of autophagy leading to gastric cancer

Dysregulation of autophagy during *H. pylori* infection undoubtedly exacerbates the progression from gastritis to gastric cancer. For example, cagA and vacA can inhibit the activation of upstream signaling pathways of autophagy and thus inhibit autophagy of gastric mucosal cells in precancerous lesions of gastric cancer (Zhang et al., 2022b). The signaling pathways that link gastric cancer to *H. pylori* infection and are mediated by autophagy form a multi-layered protein network for regulation. Different signaling pathways have different functions and can interact with each other. Activation or abnormal induction of one of these pathways can lead to a cascade of cellular immune damage, ultimately culminating in the development of gastric cancer (Yang et al., 2022). For example, *H. pylori* can activate NF-κB and autophagy through nucleotide-binding oligomerization domain 1 (NOD1), allowing the bacterium to persist in the gastric niche and cause carcinogenic consequences (Suarez et al., 2015). In particular, *H. pylori* and its virulence factors can disrupt autophagy, leading to increased EMT (Shi et al., 2019). The occurrence of EMT and chronic inflammation leads to the emergence of cells with tumor stem cell (CSC) characteristics, such as the ability to migrate, invade and form tumor spheres (Courtois et al., 2021). In addition, the complex process of carcinogenesis triggered by *H. pylori* is closely linked to the genetic background of the host. Many of these genes influence cellular signaling pathways that contribute to inflammatory signals, inflammasome formation and autophagy (Mommersteeg et al., 2018). For example, clinical studies have shown that the autophagy gene ATG16L1 (rs2241880, G allele) was genotyped in subjects from different ethnic cohorts (Dutch and Australian) with premalignant gastric lesions of varying severity. The mechanism of the increased risk associated with ATG16L1 rs2441880 could be attributed to its modulation of *H. pylori*-induced ER stress signaling pathways and pro-inflammatory mediators (Mommersteeg et al., 2022).

### Ferroptosis and *H. pylori* infection

Ferroptosis is a form of regulated cell death that is characterized by iron-dependent lipid peroxidation and differs from other forms of programmed cell death such as apoptosis and pyroptosis. This process was first described in 2012 and has since attracted considerable attention due to its role in various physiological and pathological conditions (Dixon et al., 2012). At the molecular level, ferroptosis involves the accumulation of lipid hydroperoxides, particularly phospholipids containing polyunsaturated fatty acids (PUFAs), resulting from the dysregulation of cellular antioxidant systems, including glutathione peroxidase 4 (GPX4) and the cystine/glutamate antiporter system xCT (Stockwell, 2022). GPX4 is a key enzyme responsible for the reduction of lipid hydroperoxides to non-toxic lipid alcohols, thus protecting cells from oxidative damage. Inhibition or depletion of GPX4 leads to an accumulation of lipid peroxides and ultimately triggers ferroptotic cell death (Yang et al., 2014). Several signaling pathways and molecules are involved in the regulation of ferroptosis. For example, the tumor suppressor p53 and the nuclear factor erythroid 2-related factor 2 (Nrf2) have been shown to modulate ferroptotic cell death through their effects on lipid peroxidation and antioxidant defense. In addition, proteins related to iron metabolism, such as ferritin, transferrin and TfR1, play a crucial role in regulating intracellular iron levels and thus influence susceptibility to ferroptosis (Stockwell, 2022).

Infection with *H. pylori* has a considerable influence on the process of ferroptosis (**Figure 3**). *H. pylori* and its components reduced the expression of LPCAT3, which plays a role in the generation of the lipid peroxide substrate. They also downregulated genes involved in iron uptake, such as TfR1. In addition, they upregulated the cystine/glutamate antiporter subunit SLC3A2 to counteract glutathione depletion, which attenuates ferroptosis (Melo et al., 2024). *H. pylori* infection can also influence the development of gastric cancer by affecting ferroptosis-related genes. Suppressor of cytokine signaling 1 (SOCS1), which is known to be a driver of ferroptosis, showed significant upregulation in both *H. pylori*-infected individuals and patients with gastric adenocarcinoma (STAD). Furthermore, increased SOCS1 expression correlated with an unfavorable prognosis in STAD patients. The increase in SOCS1 was associated with increased infiltration of immune cells and upregulation of immune checkpoints in STAD patients (Yan et al., 2023). Liu et al. demonstrated that the ferroptosis-related gene YWHAE is highly expressed in both *H. pylori*-associated gastritis and gastric cancer. The expression of YWHAE positively correlates with ferroptosis in gastric cancer and is associated with several cancer-related signaling pathways, including MAPK, NF-κB and PI3K (Liu et al., 2023). Zhu et al. also demonstrated that the molecular subtypes regulated by ferroptosis-associated genes correlate with TME cell infiltration and *H. pylori* infection increases the susceptibility of gastric cancer cells to RSL3-triggered ferroptosis (Zhu et al., 2023). To sum up, *H. pylori* appears to trigger pathways that can either enhance or block ferroptosis.

## Therapy associated with PCD

Triple or quadruple therapies are the first choice in the treatment of *H. pylori* infections. Although conventional clinical therapies can effectively kill *H. pylori*, the continuous increase in antibiotic resistance has led to a decrease in the efficacy of standard triple and quadruple therapies. In addition, side effects related to *H. pylori* eradication, such as diarrhea, taste disturbance and nausea, have a certain incidence rate among patients (Liang et al., 2022). However, PCD such as autophagy, can play a role in eliminating intracellular *H. pylori* (Huang et al., 2018). With the increasing research on *H. pylori* infection related to PCD dysregulation, exploring the mechanisms of action of *H. pylori* virulence factors and their major targets in the different pathways of PCD may become an important research direction for future treatments of *H. pylori* infection and *H. pylori*-related gastric cancer. Establishing animal and cell models of *H. pylori* infection, exploring different cell death signaling pathways and gaining deeper insights into their importance in the pathogenesis of the disease, and searching for biologically active substances that can inhibit or induce PCD may be crucial for future treatments of *H. pylori* infection and associated gastric cancer (**Table 2**).

**Table 2:** Biologically active compounds targeting cell death pathways in *H. pylori* infection.

Programmed cell death	Compound	Mechanism of action	References
Apoptosis	Tanshinone IIA Quercetin L-ascorbic Acid-2-Glucoside Eudesmin Lactobacillus rhamnosus JB3	Increases apoptotic relevant protein BAX and caspase-9 expressions in *H. pylori* infection. Affects the levels of BCL-2 and BAX to protect against apoptosis associated with *H. pylori* infection. Improves mitochondrial function by restoring the level of ATP and MMP, inhibits *H. pylori* induced apoptosis. Suppresses the activation of apoptosis associated proteins induced by *H. pylori*. Inhibits intrinsic apoptosis proteins including BAX, cytochrome c, and caspase-3 mediated by infection.	(Chen et al., 2016) (Zhang et al., 2017c) (Chen et al., 2018) (Yang et al., 2018) (Do et al., 2021)
Autophagy	Chloroquine Glycyrrhizin Simvastatin Astaxanthin	Inhibits the increased expression of Beclin1 and LC3B-II, thereby reducing autophagy in *H. pylori* infection. Restores autolysosomal function by inhibiting HMGB1 to ameliorate *H. pylori* infection. Enhances early endosome maturation and subsequently activates the autophagy pathway. Induces autophagy through the activation of AMPK and the downregulation of its downstream target, mTOR.	(Li et al., 2023) (Khan et al., 2023) (Liao et al., 2016) (Lee et al., 2020)
Pyroptosis	Rabeprazole	Inhibits pyroptosis by alleviating GSDMD-executed pyroptosis, leading to decrease IL-1β and IL-18 mature and secretion.	(Xie et al., 2021)

ATP, Adenosine triphosphate; MMP, Mitochondrial membrane potential; HMGB1, High mobility group box 1; AMPK, Adenosine monophosphate-activated protein kinase; mTOR, Mammalian target of rapamycin.

During apoptosis, several biologically active substances act in different phases, mainly influence intrinsic apoptosis. During the initiation phase of intrinsic apoptosis, upregulated BCL-2 homology region 3 proteins bind to survival-associated BCL-2 proteins and release BAX and BAK from their inhibition by survival-associated BCL-2 proteins. Quercetin modulates the balance between proliferation and apoptosis of gastric cells by affecting the levels of BCL-2 and BAX, thereby downregulating apoptosis induced by *H. pylori* infection and exerting a protective effect against gastritis-associated inflammation (Zhang et al., 2017c). In the subsequent steps of intrinsic apoptosis, BAX and BAK form oligomers that trigger mitochondrial events. L-ascorbic acid-2-glucoside (AA2G) reduces *H. pylori*-induced cell apoptosis by modulating the mitochondria-dependent apoptotic signaling pathway. The mechanism may be related to the restoration of mitochondrial ATP level and membrane potential, thereby improving mitochondrial function to protect gastric epithelial cells from *H. pylori* infection (Chen et al., 2018). After the mitochondrial phase, the assembly of the apoptosome scaffold is triggered and the caspase process is activated. Eudesmin inhibits the growth of *H. pylori* and suppresses the activation of caspase-associated proteins (caspase-3, -8, -9) induced by *H. pylori* (Yang et al., 2018). There are also preparations that can regulate intrinsic apoptosis in multiple steps, such as Lactobacillus rhamnosus JB3, which can dose-dependently inhibit proteins involved in the intrinsic apoptotic pathway, including BAX, cytochrome c and caspase-3, thus suppressing cell apoptosis induced by *H. pylori* (Do et al., 2021). The above preparations can all reduce the abnormal increase in apoptosis caused by *H. pylori* infection. However, in the chronic stage of *H. pylori* infection, apoptosis often decreases (Nagy et al., 2009). In chronic infection, treatment with tanshinone IIA significantly increases the expression of apoptosis-related proteins BAX and caspase-9, disrupts mitochondrial transmembrane potential, triggers the release of cytochrome c and activates caspase cascades. It markedly promotes intrinsic cellular apoptosis via the NF-κB and MAPK pathways and exerts a protective effect on host cells in severe inflammation and *H. pylori*-induced gastric cancer (Chen et al., 2016).

In autophagy, *H. pylori* infection can disrupt lysosomal function, leading to the development of enlarged and dysfunctional lysosomes and autophagosomes (Capurro et al., 2020). At the same time, infection leads to upregulation of autophagy-related proteins Beclin1 and LC3B-II, while chloroquine can inhibit this abnormal autophagic expression (Li et al., 2023b). In addition to suppressing abnormal autophagy during *H. pylori* infection, bioactive compounds can also promote the development of beneficial autophagy. The lysosomal autophagy pathway was impaired by an increase in lysosomal membrane permeabilization during *H. pylori* infection. However, glycyrrhizin preserves the integrity of the lysosomal membrane, which in turn facilitates the formation of autolysosomes. This restoration of lysosomal function leads to a reduction in intracellular *H. pylori* growth by eliminating the pathogenic niche (Khan et al., 2023). In addition, simvastatin can promote early endosome maturation and subsequently activate the autophagy pathway, which promotes lysosomal fusion and leads to the degradation of sequestered bacteria, thereby alleviating *H. pylori*-triggered inflammation (Liao et al., 2016).

Biologically active compounds can also comprehensively regulate several PCD processes to reduce the damage to normal PCD in the body caused by *H. pylori* infection. For example, astaxanthin induces autophagy by activating AMPK and downregulating its downstream target mTOR. When astaxanthin levels increase, it can also inhibit cell apoptosis induced by *H. pylori* (Lee et al., 2020). In addition, we should emphasize the use of the proton pump inhibitor rabeprazole in triple or quadruple drug regimens. In contrast to conventional acid-suppressive and antimicrobial drugs, rabeprazole acts by modulating PCD (Xie et al., 2021). NLRP3 and GSDMD are significantly increased in the gastric tissue of patients with *H. pylori* infection. Lansoprazole can attenuate GSDMD-induced cell pyroptosis, significantly inhibit the expression of ASC, NLRP3 and caspase-1, and thus lead to a reduction in the maturation and secretion of IL-1β.

Therapeutic interventions targeting regulators and effectors of various cell death pathways hold promise for improving treatment outcomes in patients with *H. pylori* infection. Given the complex nature of *H. pylori* infection, in which multiple cell death mechanisms interact with other cellular processes, effective therapies will likely include combinations of agents targeting different cell death programs, as well as molecules that affect additional cellular pathways. Therefore, the efficacy of the above agents at the animal and cellular level is promising, but their clinical utility needs to be further explored.

## Discussion

A comprehensive understanding of programmed cell death has revealed ways to address aberrant situations in *H. pylori* infection. In apoptosis, *H. pylori* infection can cause dynamic changes in apoptosis levels in different cells. The increase in apoptosis in the acute phase may be an important mechanism by which the bacteria damage the stomach, while the decrease in apoptosis in the chronic phase leads to the formation of a tumor microenvironment that promotes the development of gastric cancer. In pyroptosis, the signaling pathway most closely associated with *H. pylori* infection is NLRP3. During infection, the pyroptotic inflammasome is susceptible to external stimuli such as microbes, environmental factors or inflammasome activators, leading to abundant production of inflammatory factors and promoting the occurrence of human gastric tumors. In autophagy, *H. pylori* and its virulence factors disrupt the normal level of autophagy, leading to an accumulation of damaged cellular components in the early stages and triggering autophagic cell death. In the later stages of infection, however, autophagic function is impaired, eventually leading to the development of gastric cancer. As for necroptosis and ferroptosis, there is relatively little research on their processes in *H. pylori* infection. However, animal studies, cell experiments and clinical observations have confirmed the importance of these two forms of cell death in *H. pylori* infection, and further investigation of their interaction is warranted.

The key question is whether blocking or promoting a specific PCD signaling pathway would be beneficial for the clinical treatment of *H. pylori* infection and associated gastric cancer. The prospects of such a therapy should be further investigated. We hope that research on PCD in the context of *H. pylori* infection will continue to progress and eventually lead to valuable life-saving treatments.

## Author contributions

YL: Writing – original draft, Writing – review & editing. KL: Writing – original draft, Writing – review & editing. FL: Data curation, Visualization, Writing – review & editing. CZ: Conceptualization, Funding acquisition, Writing – review & editing. FC: Conceptualization, Writing – review & editing.
